# Patient Uptake, Experiences, and Process Evaluation of a Randomized Controlled Trial of Internet-Based Cognitive Behavioral Therapy for Tinnitus in the United States

**DOI:** 10.3389/fmed.2021.771646

**Published:** 2021-11-17

**Authors:** Eldre W. Beukes, Gerhard Andersson, Vinaya Manchaiah

**Affiliations:** ^1^Department of Speech and Hearing Sciences, Lamar University, Beaumont, TX, United States; ^2^Vision and Hearing Sciences Research Centre, School of Psychology and Sport Sciences, Anglia Ruskin University, Cambridge, United Kingdom; ^3^Virtual Hearing Lab, a Collaborative Initiative Between Lamar University, Beaumont, TX, United States, and the University of Pretoria, Pretoria, South Africa; ^4^Department of Behavioral Sciences and Learning, Department of Biomedical and Clinical Sciences, Linköping University, Linköping, Sweden; ^5^Division of Psychiatry, Department of Clinical Neuroscience, Karolinska Institute, Stockholm, Sweden; ^6^Department of Speech-Language Pathology and Audiology, University of Pretoria, Pretoria, South Africa; ^7^Department of Speech and Hearing, School of Allied Health Sciences, Manipal Academy of Higher Education, Manipal, India

**Keywords:** process evaluation, clinical trial, internet-interventions, tinnitus, cognitive behavioral therapy, digital therapeutics, experiences, patient uptake

## Abstract

**Introduction:** An internet-based cognitive behavioral therapy (ICBT) offers a way to increase access to evidence-based tinnitus care. To increase the accessibility of this intervention, the materials were translated into Spanish to reach Spanish as well as English speakers. A clinical trial indicated favorable outcomes of ICBT for tinnitus for the population of the United States. In view of later dissemination, a way to increase the applicability of this intervention is required. Such understanding is best obtained by considering the perspectives and experiences of participants of an intervention. This study aimed to identify the processes that could facilitate or hinder the clinical implementation of ICBT in the United States.

**Methods:** This study evaluated the processes regarding enrolment, allocation, intervention delivery, the outcomes obtained, and the trial implementation. The study sample consisted of 158 participants who were randomly assigned to the experimental and control group.

**Results:** Although the recruitment was sufficient for English speakers, recruiting the Spanish participants and participants belonging to ethnic minority groups was difficult despite using a wide range of recruitment strategies. The allocation processes were effective in successfully randomizing the groups. The intervention was delivered as planned, but not all the participants chose to engage with the materials provided. Compliance for completing the outcome measures was low. The personal and intervention factors were identified as barriers for the implementation whereas the facilitators included the support received, being empowering, the accessibility of the intervention, and its structure.

**Conclusion:** An understanding regarding the factors contributing to the outcomes obtained, the barriers and facilitators of the results, engagement, and compliance were obtained. These insights will be helpful in preparing for the future dissemination of such interventions.

**Clinical Trial Registration:** www.ClinicalTrials.gov, identifier: NCT04004260. Registered on 2 July 2019.

## Introduction

Tinnitus is a chronic symptom, characterized by the perception of sounds in the ears or head of an individual without any external sound source, is a highly prevalent symptom affecting at least 10–15% of the adult population (1). Although not everyone is bothered by tinnitus, a proportion of those experiencing tinnitus finds it very distressing and it may affect many aspects of daily life, such as sleeping and concentrating. As experiencing tinnitus is associated with an increased risk of psychological difficulties, such as anxiety, depression, those distressed require interventions to help them cope with the tinnitus (2, 3). Managing tinnitus can be, notoriously challenging as there is often no medical cure (4). Management thus focuses on the address associated hearing loss, educating the patients, and provide tools and strategies to manage the tinnitus and associated problems. The intervention with the strongest research evidence according to tinnitus practice guidelines (5–7) and several systematic reviews (8, 9) is cognitive behavioral therapy (CBT).

A CBT is a psychological treatment addressing the unhelpful behaviors, thought patterns, and emotional reactions caused by tinnitus (10). To increase access to the CBT for tinnitus, an internet-based CBT for tinnitus (ICBT) was developed in Sweden (11) in a self-help format with psychological guidance. This program was later translated to German (12) and English (13). To further increase the accessibility, the ICBT for tinnitus is adapted to be delivered by the audiologists (14) with some training to handle the CBT elements without compromising the outcomes (15–18). To further increase the availability of CBT, the intervention was adapted for the population of the United States (19) and also translated into Spanish to reach the Spanish and English speakers (20). As a pilot study indicated the feasibility of the intervention (21, 22), a randomized clinical trial (RCT) was undertaken (21, 23). The studies in the United Kingdom were the first in which the ICBT was delivered by an audiologist (16–18). When delivered by an audiologist, this RCT in the United Kingdom indicated that ICBT led to a greater reduction in tinntius distress compared with weekly monitoring with an effective size of *d* = 0.46 [0.14–0.77]. The results were in line with the outcomes obtained in the studies in which psychologists had provided the guidance. In addition, there was a greater reduction in the negative tinnitus cognitions and insomnia. The results remained stable over the 2 month follow-up period. Although the favorable outcomes were obtained, there were some difficulties encountered during the running of the trial, largely surrounding low engagement, and the poor compliance rates for the questionnaire completion. To increase the applicability of the intervention and to prepare for later dissemination, an understanding is needed regarding the factors contributing to the outcomes, engagement, and compliance. Such understanding is best obtained by considering the perspectives and experiences of the participants of an intervention.

The process evaluations are a means of providing a framework for analyzing the key components in the healthcare interventions. Such evaluations are important as various external factors can affect the health conditions and intervention uptake (24–26). The different process evaluation models for healthcare interventions have thus been developed, such as the Reach, Effectiveness, Adoption, Implementation, and Maintenance framework [RE-AIM (27, 28)] and the components suggested by Baranowski and Stables (29) and Linnan and Steckler (30). Although each model is unique, the overlapping component includes investigating the recruitment procedures, the context of the research, the intervention delivery and how it was received, the outcomes obtained, and the implementation of the intervention. Despite the relevance of process evaluations, they are not widely used within audiology with only a few process evaluations related to hearing difficulties (31–33) and one related to the ICBT for the population of the United Kingdom (34).

The research objective of the present study was to identify the processes that could facilitate or hinder the clinical implementation of ICBT for tinnitus in the United State (23). This was done by considering the full trial implication from the recruitment to post-intervention follow-up. This was in view of gaining insights into the applicability of ICBT for the population in the United States and identify the factors that could help optimize dissemination. The specific objectives were to evaluate the processes regarding the enrollment, allocation, intervention delivery, the outcomes obtained, and the trial implementation.

## Materials and Methods

### Research Design

This study was a process evaluation of an RCT of ICBT for tinnitus conducted between March 2020 and July 2020. The process evaluation was conducted in parallel to a clinical trial that investigated the efficacy of ICBT for tinnitus in the United States (23). The participants were randomized with a 1:1 allocation ratio to the *experimental group* to receive ICBT for 8 weeks, or the *control group* who received the intervention after a delay of 8 weeks during which time they were monitored weekly. The outcome measures were completed at baseline, T1 (post-intervention for the experimental group), T2 (post-intervention for the control group), T3 at 2 month follow-up post-intervention for each group, T4 at 1 year follow-up.

The RCT and its protocol were pre-registered at the Clinical Trials.gov: NCT04004260 on July 2, 2019. Ethical approval was obtained from the Institutional Review Board at Lamar University, Beaumont, Texas, United States (IRB-FY17-209). The study was conducted and reported according to the Consolidated Standards of Reporting Trials (CONSORT) EHealth guidelines (35). An independent data monitoring committee monitored the running of the trial.

### Participants

#### Target Recruitment

Following the sample size calculations, the goal was to enroll 152 participants. To ensure inclusivity, the aim was to recruit 48 Hispanic or Latino participants and 94 non-Hispanic or Latino. The racial categories targeted were American Indian/Alaskan Native (2), Asian (6), Black or African American (18), more than one race (20), and White (106).

#### Eligibility Criteria

Eligibility was determined in a two-stage process. The inclusion criteria were that the participants needed to be aged 18 years or over and living in Texas, United States. Computer and internet access were required. The participants had to have experienced tinnitus for a minimum duration of 3 months and have a score of 25 or above on the Tinnitus Functional Index (TFI), suggesting a need for tinnitus care (36). The exclusion criteria were indications of significant depression (≥15 scores) on the Patient Health Questionnaire [PHQ-9 (37)]. Other aspects that resulted in the exclusion are: indications of self-harm thoughts or intent (i.e., answering affirming on Question 10 of the PHQ-9 questionnaire), reporting any major medical, psychiatric, or mental disorder which may hamper commitment to the program or tinnitus as a consequence of a medical disorder still under investigation.

#### Eligibility Screening

Initially, the participants completed the baseline measurements online (T0). Following completion, a telephonic screening was arranged, to ensure participants fulfilled the study requirements. For any participant indicating possible self-harm thoughts or significant depression on the PHQ-9, a psychologist would phone them within 24 h. A clear protocol was set up for these participants. The scores were discussed, and the participants were questioned as to whether they had additional help and support for these problems. If there were any concerns, a stabilization plan was set up. If this was not possible, the crisis team would be contacted. The person would be kept on the phone until the crisis team arrived, although no such cases were reported in the current study. Everyone who called on the phone was provided with the emergency contact details. The psychology or other appointments were arranged as appropriate, or referrals made were indicated.

### Intervention

The ICBT content was based on a Swedish CBT self-help program (38), transformed into an 8 week interactive e-learning version (39) and then, adapted linguistically, culturally, and functionally to ensure the suitability for the population of the United States (19, 20). The ICBT platform consisted of 22 modules with worksheets and quizzes (14). The participants were asked to read the modules weekly and ideally spend at least 10 min each day practicing the suggested strategies. The intervention specifically targeted reducing the activity limitations and participation restrictions and included applied relaxation due to the importance of this aspect in tinnitus managment (40). Both the groups received the same intervention, only the timings regarding receiving the intervention varied.

The guidance was provided to support the participants while undertaking the intervention. This included monitoring progress, monitoring the weekly scores, providing feedback on the worksheets completed, outlining the content of new modules, and answering questions. The participants who did not engage were contacted to support participation and to discuss the possible barriers. An encrypted 2-way messaging system within the ePlatform was used to communicate (39). The intervention was provided free of charge and the participants could continue to access it after the intervention was completed.

### Parameters Used for the Process Evaluation of the Clinical Trial

The overlapping and relevant elements from the healthcare process evaluation models were used to identify the process to evaluate for this clinical trial from the RE-AIM model (27, 28), from Baranowski and Stables (29) and Linnan and Steckler (30). Five processes were selected, namely, enrollment, allocation, intervention delivery, the outcomes obtained, and the trial implementation as illustrated in Table 1. A demographic questionnaire was used to establish the health-related and tinnitus-specific information at baseline (T0). The standardized outcome measures were completed at baseline (T0), after the experimental group completed the intervention (T1) after the control group completed the intervention (T2), at 2 month follow-up (T3), and 1 year follow-up (T4). The primary outcome measure was tinnitus severity as measured by the TFI (36). The secondary outcomes were:

▪ The Generalized Anxiety Disorder−7 [GAD-7 (41)] to assess the symptoms of generalized anxiety disorder.
▪ The PHQ-9 (37) (38) indicated the symptoms of depression.
▪ The Insomnia Severity Index [ISI (42)] assessed the presence of insomnia.
▪ The Tinnitus Cognitions Questionnaire [TCQ (43)] was used to measure the negative tinnitus cognitions.
▪ The EQ-5D-5L (44) measured general health-related quality of life.
▪ The Tinnitus and Hearing Survey [THS (45)] was used as a short measure to identify the tinnitus severity, hearing disability, and hyperacusis of the participants.
▪ A short questionnaire was administered to try to determine the effect of coronavirus disease 2019 (COVID-19) on the study asking whether the participants had COVID-19 and how this was affecting them. This questionnaire was added during the middle of the study due to the study being administered during the height of the first wave of the pandemic.
▪ A satisfaction questionnaire was designed to assess the suitability, content, usability, presentation, and exercises from the intervention consisting of 15 five-point Likert-type scaled questions (39).
▪ The open-ended questions to find out more about the experiences from the intervention such as which modules were helpful, what barriers were found, and suggestions for improvements. This was done by both asking open-ended questions in a survey as well during the phone calls made to the participants post-intervention.

**Table 1:** The processes in the clinical trial.

**Process**	**Sub-process**	**Description**	**Data Collection**
Enrolment	Recruitment	Processes involved in approaching and attracting participants	• Evaluation of the recruitment formats used • Use of Google analytics to examine recruitment trends
	Participant screening	Processes involved in selecting the participants for the study	• Scrutiny of the inclusion criteria • Motivation ratings on a Likert scale of 1–10 • Expectations ratings on a Likert scale of 1–10 • Two-step process • Protocols
Allocation	Reach: Number recruited	Whether the target number of participants were obtained for the study and whether they represented the target population of those with distressing tinnitus	• Comparison of the recruitment targets set and achieved
	Context: Participant characteristics	The social, demographic, and socio-economic characteristics of the participants that may affect generalizability of the outcomes	• Considering participant demographical profiles (gender, age, tinnitus duration, previous tinnitus treatments) • Internet proficiency
	Randomization	The effectiveness of the randomization process selected	
Intervention delivery	Dose delivered	The amount and content of the intervention	• Number of modules • Number of videos • Guidance received
	Dose received	Participants engagement with the intervention	• Number of logins • Modules that were opened • Module ratings • Worksheets completed • Messages sent • Time spent on the modules
Outcomes	Adherence	Participants completing the outcome measures	• Percentage completing the outcome measures at each time point
	Primary outcome results	Whether tinnitus severity decreased	• The effect of the intervention on tinnitus severity
	Secondary outcomes	Whether tinnitus comorbidities improved	• Data monitoring • The effect of the intervention on anxiety, depression, insomnia, quality of life and hearing-related outcomes
Trial implementation	Implementation Fidelity	The degree to which the protocol was carried out as intended	• Comparison of the actual programme to the protocols described
	Barriers to implementation	Processes that were barriers to the implementation	• Satisfaction questionnaire • Qualitative data from participant interview • Qualitative data from open ended questions • Qualitative data from *ad hoc* messages
	Facilitation of effectiveness	Processes that facilitated effectiveness from the participants perspectives	• Satisfaction questionnaire • Qualitative data from participant interview • Qualitative data from open ended questions • Qualitative data from *ad hoc* messages

### Data Analysis

Data analysis incorporated a mixed approach, including both quantitative and qualitative analyses. The Statistical Package for Social Sciences (IMB SPSS for Windows V.26.0, NY, USA) was used for the statistical analyses (46).

Descriptive statistics were used to describe the sample characteristics. The continuous variables were summarized with means and SDs. The categorical variables were described using frequencies and percentages. The effect sizes were used to determine the outcome effectiveness. The outcomes related to the satisfaction of the intervention and the specific components were rated on a 5-point Likert scale and were analyzed using descriptive statistics.

The open-ended questions were analyzed using a qualitative content analysis described by Graneheim and Lundman (47). The content analysis enables the systematic interpretation of the participant statements to identify the central aspects (a set of condensed categories) that emerge from careful examination of the raw data using a bottom-up approach. Various steps were involved in the process. Initially, the responses were read repeatedly and coded for “meaning units,” which are statements that relate to the same central category. These meaning units formed the units of analysis for coding. The next process was identifying the categories that were repeatedly mentioned. The responses that related to the same category were grouped together. The repeated patterns were further grouped until the clear condensed categories and subcategories were identified. The codes were then gradually merged into the broader categories and subcategories by grouping thematically similar codes together. The categories were subsequently condensed by combining the categories with similarities, ensuring that the categories were mutually exclusive. The category labels were assigned. After selecting the codes and categories, the original responses were checked to ensure they were in line with the assigned categories and to identify if any additional categories emerged. The dataset was rechecked for consistency. The data coding was performed independently by the two researchers. The coding was compared and in case of inconsistencies, these were discussed.

## Results

### Processes Related to the Enrollment

The recruitment and participant screening processes are discussed in this section.

#### Processes Related to Recruitment

The multidimensional recruitment strategies were employed to obtain a varied sample. Table 2 outlines the different recruitment strategies used and how successful each was. A comprehensive study website was designed to provide information for those who were interested in the study. This included how to register, the aims of the program, the time commitment, and the nature of the intervention. All the recruitment strategies guided individuals to the study website (www.tacklingtinnitus.org). Google analytics indicated that 3,720 users viewed the website as outlined in Table 2. This indicates that the recruitment strategies drew sufficient interest to the website.

**Table 2:** The various recruitment strategies used.

**Recruitment Means**	**Reach**	**Facilitators**	**Barriers**
Intervention website (www.tacklingtinnitus.org)	• 3,720 users • Average pages viewed: 11 • Average session duration: 10 min • 24% returned; 76% were new • Peak in views were during March 2020 during the recruitment period • 71% found the website directly, 19% via social media, 8% *via* a search engine, 2% via referral	• Information in English and Spanish • Cost-effective • Informative, all the information in one place • Gives a feel of the intervention as the same website pages are used	• Difficult to attract only participants meeting the inclusion criteria • Recruitment pages were long. They may be too long and put people off
Location of website views	United States *n* = 3,720; 82% Spain *n* = 141, 4% Mexico *n* = 88; 3% Argentina *n* = 48, 1%	Attracted mostly from the United States as required	• Difficult to target only those in Texas (attracted 18% from other countries countries)
Story board YouTube video	Premiered before the pilot study on September 21, 2019 and had 549 views at the time investigation	Attractive, information presented in an auditory and visual format in English and Spanish	• Very costly
Professional recruitment agency (i.e., Trial Facts)	Number recruited: 92 (44 English, 38 Spanish) Online screening: 25 not suitable Phone screening by study team for: 67 Phone screening indicated unsuitability: 18 not suitable/ not interested/ or not answering calls Passed screening: 49 Of those passing, 23 enrolled in the program (25% enrolled)	• Clear outline provided of the reasons people did not meet the inclusion criteria • Clear processes to follow	• Very costly • Additional screening processes requiring additional time resources from the study team required for these participants • Recruitment materials needed to be provided and formatted by study team for this company requiring additional time • Feedback had to be provided to the company using their software • Additional time with meetings for the agency • Recruitment of Spanish and ethnic minorities numbers still low
Direct contact	Leaflet/ posters to churches, old age homes, community centers to target Spanish participants and ethnic minorities in particular	Reaching people who may not find out via the internet or social media	• Poor understanding of the Spanish publics' perspective of recruitment • Costly • Needed a lot of time from the study team
Targeting those patients seeking help	Emails/ posters/ leaflets provided to professionals who may see patients with tinnitus asking them to pass on • Audiologists • Psychologists • Physicians/ doctors • Ear, Nose and Throat (ENT) specialists • Student clinics at Lamar University	Reaching people with probably bothersome tinnitusBuilding networks with the professional community	• Costly • Needed a lot of time from the study team
Public Patient Initiative (PPI)	• Used to gain ideas for recruitment • The contacted their local professionals	• Participant perspectives helpful • Video of experiences	Could have involved more in all processes during recruitment
Media	• Press release • Television advertisement • Radio advertisement • Newspapers (Beaumont enterprise)	Many readers with potential for a good reach	• Very costly • Difficult to find contacts
Social Media	• Twitter • Instagram • YouTube • Facebook	Potential for a good reach	Needed additional resources from the study team to set up and manage
Patient organizations	• American Tinnitus Association (ATA) • Sertoma, Spanish organization helping people with hearing loss	Building networks with these organizations	Could not control how much/ little the distributed the information
Tinnitus support groups	• The researchers, audiologists and member from the public patient involvement group attended tinnitus support groups to share information about the intervention and encourage recruitment	• The face-to-face contact was appreciated • Recruitment strategy was effective	• Costly • Time consuming • Many of these people are already helped and don't need the intensity of such a program

All the recruitment materials were translated from English into Spanish, adding an extra layer of complexity. Both the English and Spanish team members were required for this study. Recruiting the Spanish participants required additional thought and it was difficult to target these participants. Direct contact was included during the recruitment, using a public patient initiative (PPI).

#### Processes Related to Participant Screening

**Figure 1:**
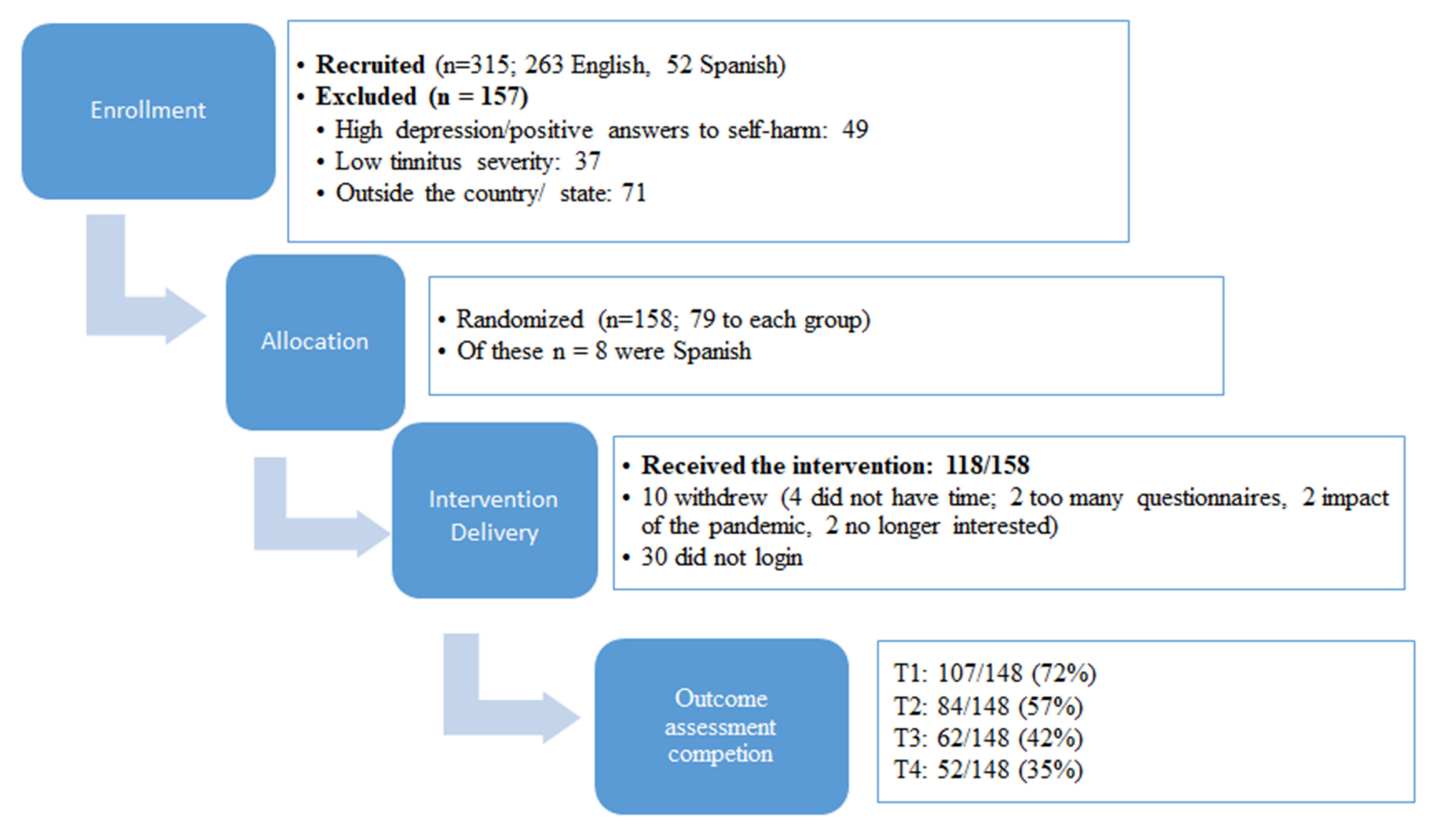
Flowchart of enrollment, allocation, intervention delivery, and outcome assessment completion. T1 is after the experimental group received the treatment, T2 after the control group received the treatment, T3 is at 2 month follow-up, and T4 is at 1 year follow-up.

There were 157 participants who registered on the study website and showed interest prior to the recruitment opening. Further recruitment means drew a total of 315 participants who showed interest in the study and were screened (263 English and 52 Spanish). Of these, 158 were eligible (as shown in Figure 1). The exclusion reasons included having high depression scores or a positive answer regarding self-harm intent (46 English and 3 Spanish), low tinnitus severity (36 English and 1 Spanish), living outside the recruitment area in the State of Texas, United States (5 English and 40 Spanish). According to the protocol set for this trial, a psychologist was required in the team to make phone calls to the 49 participants (three using translation) who had high depression scores. Having a psychologist was helpful, as tinnitus is best approached from a multidisciplinary perspective (4–10) but involving more experts may be an expense, not all the teams can accommodate. As all the participants contacted were those with known depression that was being treated and there were no cases that raised concern, this particular study did not specifically require the expertise of the psychologist to deal with any serious depression or self-harm intent.

### Processes Related to Allocation

#### Reach

The target numbers according to sample size calculations were *n* = 152. A total of 158 participants were enrolled as shown in Figure 1, which indicated that the required participants were reached. There were fewer Spanish speaking participants than aimed for.

#### Context

The social, demographical, and socio-economic characteristics of the participants were identified. Equal gender ratios were recruited with *n* = 80 (51%) being female and *n* = 78 (49%) being male. A wide age range was represented (19–84 years) with a mean of 57 (SD: 12) years, which correspond to the expected range due to the incidence of tinnitus being most prevalent in the 40–70 years age range (1). A wide range of tinnitus duration was found (3 months−70 years) with the average tinnitus duration being 14 years (SD: 14).

The majority had obtained a university degree (*n* = 84; 53%) or other training vocationally or from a college (*n* = 53; 34%). Only a minority had only a high school qualification (*n* = 21; 13%). The majority were skilled workers or professionals (*n* = 95, 61%) with only 10 (6%) not working, and 52 (33%) being retired. To ensure the participants were representative of those living in the United States, different ethnic categories were targeted. In addition, the planned ethnic enrollment was less than expected as shown in Table 3. Most of the participants indicated that they were frequent computer and internet users (*n* = 144, 91%) with only 9% (*n* = 14) having only basic computer skills.

**Table 3:** The ethnic and racial characteristics of the participants.

**Ethnic Category**	**Sex/Gender**		
	**Females**	**Males**	**Total**
Hispanic or Latino	7 (29)	13 (29)	20 (58)
Not Hispanic or Latino	73 (47)	65 (47)	138 (94)
Ethnic Category: Total of All Subjects	80 (76)	78 (76)	158 (152)
**Racial Categories**
American Indian/Alaska Native	0 (1)	0 (1)	0 (2)
Asian	0 (3)	1 (3)	1 (6)
Black or African American	2 (9)	2 (9)	4 (18)
White	78 (53)	70 (53)	148 (106)
More than One Race	0 (10)	5 (10)	5 (20)
Racial Categories: Total of All Subjects	80 (76)	78 (76)	158 (152)

*Numbers in the parenthesis are the planned enrollment numbers*.

The clinical presentation of the participants indicated tinnitus severity at a level requiring the need for a tinnitus intervention with a mean TFI score of 53.98 (SD: 17.54). The mean anxiety score on the GAD-7 was 5.6/21 (SD: 4.26) and the mean PHQ-9 was 5.48/27 (4.12) indicating mild anxiety and depression. This reflects the inclusion criteria requiring no participants with significant levels of depression (15 or more on the PHQ-9). The ISI indicated that this group had subthreshold insomnia with a score of 10.05 (SD: 5.84). The context of the research thus showed that the participants with troublesome tinnitus and a wide range of demographic backgrounds were drawn to the study.

#### Randomization

As an unbiased randomization process is required in a clinical trial, the randomization process was considered. Randomization was not done by the team directly involved with the participants to avoid any possible bias. The team statistician provided computer-generated randomization scheduled and an independent research assistant randomized the participants in a 1:1 allocation in the blocks of varying sizes after the participants were pre-stratified for language (English and Spanish). Following randomization, no group differences were evident as there was no estimated difference in the baseline tinnitus severity between the groups (*p* = 0.92). The demographic profiles of the groups were similar in the terms of variables, such as gender and age. The participants and investigators could not be blinded to the group allocation due to the nature of the intervention. To minimize bias, the participants were informed when the intervention would commence but not explicitly to which group they were assigned.

### Processes Involved in Intervention Delivery

#### Dose Delivered

The intervention materials were released weekly over and 8 week period. Each week, the participants received 2–3 modules, a practice diary, and videos of the techniques as shown in Table 4. The dose was delivered as planned and according to the protocol. It outlines that a comprehensive intervention was delivered consisting of 22 modules and a variety of other elements. This included videos in most of the modules. In addition, the participants received weekly guidance in the form of messages to provide feedback on the work done and to try to encourage the participants who were not engaging during the intervention.

**Table 4:** Dose delivered and received for 118 participants undertaking the internet-based cognitive behavioral therapy (ICBT) intervention.

	**Dose delivered**	**Dose received**
Logged into the platform	158 = Login information, with reminders, phone calls and text messages to encourage login	118 of the 158 participants with an average of 8.1 logins (SD: 11.3)
Number of modules	22 (17 recommended and 5 optional) releasing 2–3 weekly	Average 6.4 (SD: 7.9)
Number of videos	16	*Positive feedback:* Expert opinions helped me give more trust to the material; very informative *Negative feedback*: Some were a little too long
Guidance received	5,660 messages (36 per average for the 158 participants)	163 messages sent (Mean 1.0, SD: 2.8)
Time spent on the modules	Materials for ~20–40 min per module depending on the content and tasks	20 min: 45/118 participants 20–45 min: 51/118 participants Longer than 45 min: 18/118 participants
Program completion		Yes: 64/118 (54%) No: 54/118 (46%)

#### Dose Received

Only 54% of the participants were able to complete the 8 week CBT course as 10 withdrew and 38 never accessed the intervention materials. Table 4 shows the extent to which participants actively engaged and interacted with the resources provided. Table 5 shows how many users opened each module. There was a steady decline from 104 opening the initial module to 30 doing the final module. The engagement for the optional modules was also low ranging between 55 and 19 openings of each module. The number of worksheets completed was reduced from 86 for the initial worksheet to 14 for the later worksheets.

**Table 5:** The engagement and satisfaction with the intervention.

**Module**	**Number of users opening the modules**	**Number of participants completing the worksheets**	**Intervention satisfaction: Scale of 1–10. Mean (SD)**			**Examples of the usability of the information**
			**Understand-ability was the module**	**Usefulness of the information**	**Applicability of the information**	
**Recommended modules**						
Introduction	104 (88%)	No 1: 86 (73%) No 2: 72 (61%)	NA	NA	NA	NA
Tinnitus overview	84 (71%)	No 1: 79 (60%) No 2: 77 (65%) No 3: 74 (63%) No 4: 72 (61%)	9.5 (1.0)	9.1 (1.2)	9.3 (1.1)	I now understand that I am somewhat in control of my tinnitus, in that I can change the way I think about it, which will change my feelings, which will change my reaction to it. Thereby taking the importance off of it, and ultimately accepting it as a part of me.
Deep relaxation	83 (70%)	No 1: 79 (60%) No 2: 76 (64%) No 3: 74 (63%)	9.4 (1.0)	9.1 (1.5)	9.0 (1.8)	Seems like a good way to stop the feedback loop of anxiety by interrupting some of the physiological practices that reinforce anxiety. The connection between anxiety and tinnitus is noticeable. I can see where this practice can help to interrupt that connection
Positive imagery	59 (50%)	No 1: 49 (42%) No 2: 47 (40%)	9.2 (1.7)	9.0 (1.7)	8.6 (2.1)	I was honestly amazed by how much I let my mind take me on a journey. I completely forgot about my tinnitus for a good chunk of it
Deep breathing	51 (43%)	No 1: 49 (42%) No 2: 43 (36%)	9.3 (1.2)	9.1 (1.5)	9.3 (1.5)	What stood out was that we typically don't get enough air with shallow breathing, and especially when we're tense. Also placing 1 hand on chest and the other on belly helps me feel the difference between chest and belly breathing
Changing views	48 (41%)	No 1: 43 (36%) No 2: 43 (36%) No 3: 39 (33%) No 4: 37 (31%) No 5: 38 (32%)	9.0 (1.4)	8.4 (2.0)	8.4 (2.1)	I thought the sounds you hear all the time you just never pay attention to, but they are there like the ceiling fan, or Ice Box. I like the idea suggested of listening to the waves but then diving in the water to make the waves less noticeable is a way of helping me to think about it all
Entire body relaxation	47 (40%)	No 1: 24 (20%)	9.7 (0.6)	9.2 (1.4)	9.3 (1.4)	I like the idea of whole body relaxation done quickly. I feel it is as or more effective than the slower way
Shifting focus	42 (36%)	No 1: 40 (34%)	9.4 (0.9)	9.0 (1.5)	9.0 (1.8)	The technique itself, shifting focus between two things and shifting focus between one object and tinnitus, is new to me. The explanation in the video about how tinnitus is not worthy of attention is quite helpful too. I think I'll start answering my tinnitus with that thought
Frequent relaxation	38 (32%)	No 1: 22 (19%)	9.8 (0.8)	9.0 (2.0)	9.0 (2.0)	I am struggling this week, just lost a good friend and it seems like the whole country is in chaos right now. But relaxation techniques are really valuable right now, not just for coping with tinnitus
Thinking patterns	39 (33%)	No 1: 30 (25%) No 2: 31 (26%) No 3: 17 (14%)	9.2 (1.0)	9.0 (1.4)	8.7 (1.5)	I am amazed that my thinking pattern is making such a havoc physical mentally and emotionally my body and nervous system is worn out from fighting myself Thankful getting some understanding and help in CBT I can see there is a light in the end of the tunnel. I have so far benefited from this program tremendously
Quick relaxation	39 (33%)	No 1: 20 (16%)	9.6 (0.7)	9.4 (1.2)	9.4 (1.1)	I didn't really consider before of doing rapid relaxation. It seems like less pressure to do rather than spending a lot of time trying to relax. I like that it is quick and easy
Challenging thoughts	38 (32%)	No 1: 14 (11%)	7.9 (2.0)	8.6 (1.9)	8.8 (1.7)	I never thought about challenging my negative thoughts, nor did I realize how different mindsets can interfere with our thinking. I recognize all of the mindsets (except for Blaming) as ones that I do a lot. My plan is to determine which mindset I'm in at the time of a negative thought and then try to switch to an opposite mindset
Relaxation routine	37 (31%)	No 1: 16 (13%)	9.7 (0.9)	9.3 (1.3)	9.3 (1.3)	Making time to enjoy things is important and is part of relaxation. The routine specified actually sounds a lot more doable than I had imagined
Being mindful	33 (28%)	No 1: 20 (16%)	9.9 (0.6)	9.1 (1.5)	9.0 (1.5)	Slowing down to focus and enjoy the moment in time helps in relaxation, my breathing, I can feel my body responding in an overall calmness
Listening to tinnitus	36 (31%)	No 1: 25 (21%)	9.9 (0.3)	9.3 (1.3)	9.2 (1.6)	This module is one of the best, only behind relaxation! I am not anxious about my tinnitus anymore. It's just a minor annoyance
Key point summary	31 (26%)	Reported elsewhere	NA	NA	NA	NA
Future planning	30 (25%)	No 1: 22 (18%) No 2: 27 (22%) No 3: 21 (17%)	NA	NA	NA	NA
Sound enrichment	55 (47%)	No 1: 17 (14%)	9.0 (0.7)	9.1 (1.9)	9.4 (1.0)	I have been trying to cover up my Tinnitus sound so I did not hear it. Now I understand that my brain has to get use to the tinnitus sound and have the masking sound just below the Tinnitus sound
Sleep guidelines	38 (32%)	No 1: 29 (24%) No 2: 28 (23%) No 3: 26 (22%) No 4: 22 (18%) No 5: 24 (20%) No 6: 16(13%)	9.7 (0.7)	9.1 (1.6)	8.6 (2.0)	Learned that our sleep cycles during the night go up/down. I also plan to implement the 20 min rule about getting up if unable to sleep after 20 min
Improving focus	28 (24%)	No 1: 11 (9%)	9.5 (0.8)	9.2 (1.4)	9.3 (1.2)	Take breaks to allow for better concentration, tinnitus is not always the reason for lack of concentration
Sound tolerance	26 (22%)	No 1: 14 (11%) No 2: 17 (14%)	9.6 (0.7)	9.8 (0.7)	9.5 (0.9)	This module was incredible. I finally feel understood and like I have been given advice I can truly implement in my life instead of just hearing “you can't do anything about tinnitus or hyperacusis”
Listening tips	19 (16%)	No 1: 5 (4%) No 2: 7 (5%)	9.9 (0.3)	9.9 (0.3)	9.8 (0.6)	I must pay better attention to the environment to modify the things I can for better hearing perception
Goals	NA	Initial: 72 (61%) Mid-program: 27 (22%) End: 22 (18%)	NA	NA	NA	NA
Practice worksheet		Completed 364 times	NA	NA	NA	NA

To identify whether the engagement was related to satisfaction with the modules, the satisfaction for each module is shown in Table 5. The overall ratings were high for being able to understand the modules at 9.4/10 (SD: 1.2), the usefulness of the information 9.1/10 (SD: 1.6), and applicability of the information was 9.0/10 (SD: 1.6). These ratings are high, indicating those that did the modules found them helpful and usable as indicated by the example of the open-ended responses about what they gained from the information.

### Processes Involved in the Outcomes Obtained

#### Adherence

Overall, the compliance for completing the outcome measures was low as shown in Figure 1. The completion rates at T1 were 72%. This decreased to 57% at T2, 42% at T3, and 35% at T4.

#### Primary Outcome Results

The main outcome was a reduction in tinnitus distress. This was achieved as indicated by an effect size of *d* = 0.46 (*CI*: 0.14–0.77) after the experimental group received the treatment (23). After the control group received the treatment, their tinnitus severity reduced. These improvements were maintained during the 2 and 12 month follow-up periods. These results were clinically significant for 51% of the participants from both the groups after completing the intervention (*n* = 75/148) indicating that their tinnitus severity reduced by more than 22.74 points. Hence, although the engagement was not optimal, the improvements in tinnitus distress were evident.

#### Secondary Outcome Results

Furthermore, the intervention led to the experimental group having a significantly greater reduction in insomnia, negative tinnitus cognitions, and hearing disability. Significant differences were not found for anxiety, depression, and quality of life, although the reductions were maintained during the follow-up periods (23).

The study reported minimal or no adverse effects. During the intervention period, only 1 (0.6%) participant had an increase of more than 10 points on the THI-S questionnaire. On finding out more, this was related to a particularly stressful deadline for work under difficult circumstances during the COVID-19 pandemic. There was only 1 (0.6%) participant who reported an adverse effect on the outcome questionnaire, explaining that initially, their tinnitus was more bothersome due to all the focus on tinnitus at the start of the intervention. There were no serious adverse events such as privacy breaches or major technical problems.

The involvement of a data monitoring committee added transparency and accountability to the results. The quarterly reports were prepared for the committee to monitor the enrollment, recruitment, results, adverse effects, and trial running.

### Processes Involved in the Trial Implementation

#### Implementation Fidelity

Various protocols were set up before commencing and a pilot trial was initially run to identify the shortcomings to aid the effective implementation of the clinical trial (21, 22). The materials were adapted to ensure they were accessible without high linguistic demands (20) and the platform was functionally acceptable (19) before running the clinical trial. The intervention was delivered between the end of March 2020 and July 2020. This was during the peak of the first wave of the COVID-19 pandemic. The intervention ran as planned, although it was started 2 weeks earlier than planned when it became apparent that the pandemic was causing disruptions to everyday life.

A questionnaire was administered to try to determine the effect of COVID-19 on the study. Only a few responses were received. Of those responses, 5/43 (12%) said that they had had the COVID-19 virus. Of those answering, 12/43 (28%) reported that the situation was affecting their tinnitus. The reasons provided included increased anxiety, stress, being more depressed, and social isolation. Almost half (21/43; 49%) indicated that the pandemic had negatively impacted their emotional state and 12/ 43 (28%) felt lonely due to the social restrictions. During the intervention, some people became ill and could not complete the program. Others were given additional time as they did not have enough energy to complete the program after recovering. Thus, the pandemic did influence the intervention fidelity for some participants.

#### Barriers to Implementation

**Figure 2:**
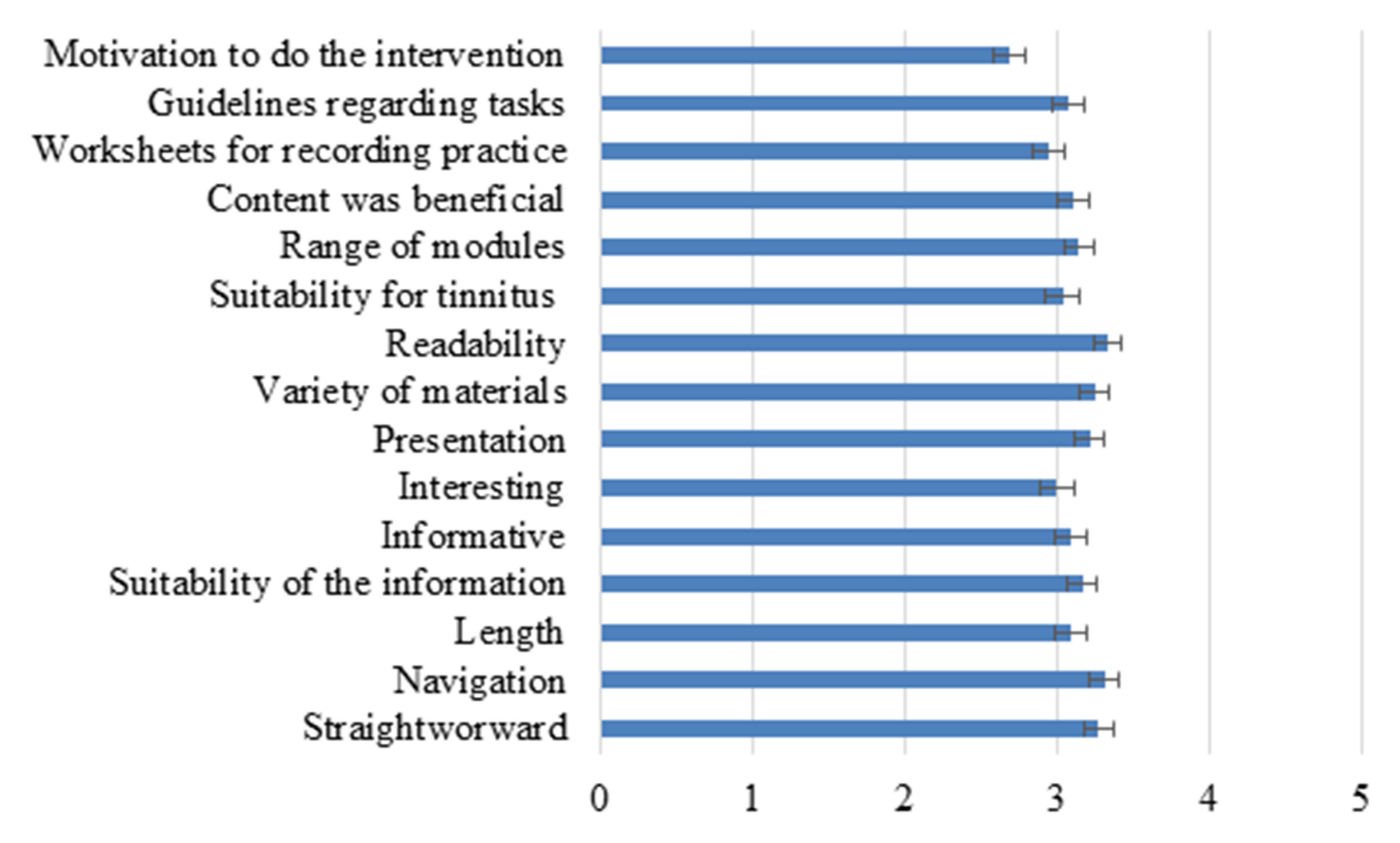
The ratings of different aspects of the intervention on a scale of 1–5. The error bars represent SEM.

To identify the barriers to intervention usage, an intervention satisfaction questionnaire (39) was completed to identify how satisfied participants were with the intervention. The mean overall score for the satisfaction questionnaire was 46/75 (61% satisfaction) which was lower than expected due to higher satisfaction during the feasibility and pilot phase (19–21). To further investigate this, the ratings for the individual questions were investigated as shown in Figure 2. The highest rating was for the readability of the materials that the navigation was clear and it was straightforward to use. The lowest ratings were for having the motivation to complete the program, the worksheets, and how interesting the information was. These intervention aspects were the barriers to the intervention engagement.

The open-ended questions were furthermore analyzed to identify the additional barriers to implementation. These included both the personal and intervention factors as shown in Table 6. The personal factors identified were time barriers, the impact of the COVID-19 pandemic, lack of self-discipline, and other health problems. The intervention factors making completion difficult included the length and number of tasks on the intervention, that tinnitus was heightened due to the focus on tinnitus during the intervention, and that some people sought a cure and not strategies to help them cope with the tinnitus.

**Table 6:** The barriers to the intervention implementation.

**Category**	**Sub-category**	**Number of meaning units**	**Example of a meaning unit**
Personal factors	Time barriers	88	“I know I must have missed some. I have been dealing with my own and my families health issues and dropped everything. including my job to deal with that, but I'm slowly going back to work”
	COVID-19 pandemic	15	“.the surge in Corona Virus cases in my area have distracted me and just make getting safely though the day challenging for the last month”
	Not using sound therapy	14	“I've been through tinnitus retraining therapy and focus on this by using sound masking for 8-12 hours a day. It is hard to fit in this time and doing the suggestions in the program without sound on”
	Health factors	9	“The fatigue and other side effects from being ill have made focusing on the program difficult”
	Lack of self-discipline	8	“Lack of self discipline…”
	Trying new techniques	6	“I'm just afraid to let go of my current method and try some new techniques in the module”
Intervention factors	Length	20	“To many modules in a very short time. I would either extent the study time or reduce the amount of modules.”
	Too many activities	2	“Too many questionnaires and worksheets for my busy schedule as a full time employee and full time caregiver.”
	Heightened awareness of tinnitus	4	“I feel like I'm focusing too much on the Demon T and I know that's not what a person is supposed to do.”
	Approach	6	“This program seemed to be more about how to cope with the tinnitus than how to get rid of it. I thought the purpose of this was to lessen the tinnitus sound more than how to cope with it”

#### Facilitation of Effectiveness

Prior to starting the intervention, the participants were asked to commit to the intervention and indicate the level of commitment on a 1–10 points scale. Those committing indicated this by a score of 10. Some found they were not able to commit as intended although this commitment motivated others as indicated by the statements, such as “I have a family and full-time job. I couldn't keep up at some point. But I made a huge commitment to the first modules (because I felt the improvement on my tinnitus) so I did my sessions every day.” This commitment by some contributed to them noticing the improvements in their tinnitus.

The open-ended responses were analyzed to identify the facilitators of effectiveness. The facilitators identified were that the intervention was empowering, accessible, well-structured, and they were adequately supported while undertaking it, as shown in Table 7.

**Table 7:** The facilitators of effectiveness to the intervention.

**Category**	**Sub-category**	**Number of meaning units**	**Example of a meaning unit**
Empowering	Gaining knowledge	51	The real insight into the condition. The knowledge base and the interplay of condition with thoughts emotions and perception. I really understood how to bring positivity into my attitude and response to my condition. Understanding the purpose/meaning behind things is helpful
	Coping techniques	13	Discovering ways to help deal with my tinnitus with actual helpful tools to cope Realizing there are techniques to reduce the impact of tinnitus and having a variety of techniques to try
	Ways of managing anxiety	18	It has helped me be less distracted and irritated with my tinnitus but I've also been able to use the technique to manage my general anxiety disorder in a more positive way
	Learning to relax	21	The techniques for calming myself in order to lessen my attention to the tinnitus were so helpful
Accessible	Flexibility	6	The convenience of being online and doing in my own schedule It can be done anywhere by yourself
Well-structured	Content	12	The modules were very well put together between the slides and the videos. The content was very relevant and made me feel like the researchers understood how patients feel about their tinnitus
	Variety	23	I appreciated how thorough and well-explained the program was presenting a wide variety of techniques as well as good solid information. There were a number of techniques shown. If one was difficult it didn't work for me i could try something else
	Well-organized	13	I have really struggled with finding good material for tinnitus. This is the most organized and helpful material that I have found. I most enjoyed the expert opinion videos and FAQs at the ends of the modules. It kept it interesting and informative with clearly defined activities and good explanations Very clear instructions and tips for practicing the different techniques and downloadable content
Support	Guidance	17	Great to have a contact at anytime when needed. My therapist was very positive and helpful throughout this experience. I appreciated the emails and calls. I could tell that my contact really cared about my condition and wanted to help

## Discussion

This process evaluation was undertaken to determine which aspects of the implementation of a clinical trial delivering ICBT to the population of the United States hampered and facilitated the outcomes obtained. The process explored included the enrollment of participants, the intervention delivery, the outcomes obtained, and trial implementation as explored in this discussion.

### Processes Involved in the Enrollment

A lot of preparation and planning was involved to ensure a range of recruitment strategies was incorporated. As this was the first tinnitus ICBT trial with Spanish participants, much research was done to investigate how to improve reaching this population [e.g., (48–52)]. The Spanish speakers were furthermore involved in the research team and during the intervention adaptation (20). Despite costly and varied recruitment strategies, it was very difficult to recruit Spanish speakers for this trial. A subsequent pilot trial with a wider recruitment area indicated that there is interest from the Spanish speakers, but ways of reaching and encouraging them to participate are still difficult (22). Moreover, although the trial targeted different ethnic and racial groups, this was not achieved and the strategies to reach a greater variety of ethnic and racial groups need to be sought.

This process evaluation highlighted various factors that could help with future trials to aid recruitment and enrollment. More hands-on involvement from a public patient group involving the individuals with bothersome tinnitus would be helpful to reach those with tinnitus (53). Such a group would advise on the strategies that the research team may not consider. Although the group members were involved in generating the recruitment ideas, directly contacting those with tinnitus at support groups, more involvement in future trials is encouraged. Hearing about the intervention effects from those with tinnitus may carry more weight than the professional contact. It was identified that a better understanding of the current public views on tinnitus and tinnitus interventions is required. Having a clear picture of what is being said in social media, public statements, on websites, on social media, blogs, and forums, advertising, policy documents, or reports provides a starting point regarding what perceptions need to be managed. Many people with tinnitus desire a treatment to completely cure tinnitus (54). Although explicitly stated that this intervention involved tinnitus management, some people still expected a cure and hence were disappointed.

It was evident that careful thought needs to be given to the inclusion criteria in the clinical trials. Excluding those with mild depression made the screening process very complicated as a psychologist had to be involved in the trial and screening process which increase the resources required. The subsequent trials indicated that including the participants with depression did not hamper the trial outcomes and their tinnitus severity decreased more than those without significant depression (23). Narrowing the recruitment to only the State of Texas was a further barrier. As this was an internet trial, using a wider pool across the country may be more helpful to reach the targeted numbers. The participants reached were those with higher socioeconomic status due to the higher levels of education. This may reflect the recruitment strategies used. An alternative way of reaching the different socio-economic groups needs to be sought which is likely to involve the alternative treatment approaches, such as less intense versions of this intervention.

### Processes Involved in the Allocation

The aim of the screening process prior to the participant allocation was to ensure that those involved were suitable for the trial, motivated to complete the intervention, and committed to completing the outcome measures for the trial. Although the participants confirmed this in the online and telephone screening, many never started the intervention. A clear need was identified to have better means of identifying who may be more engaged and motivated to do the intervention. To try to identify if this intervention is more suitable for certain tinnitus subgroups, a further trial was undertaken, dividing the participants into subgroups based on the level of their tinnitus severity (23). This indicated that the effectiveness of the intervention increased with the greater initial levels of tinnitus distress a baseline. The reductions in tinnitus distress were greater for those with significant levels of depression at the bassline. Rodrigo et al. (55) identified that the greater baseline tinnitus severity and those with greater educational levels were more likely to have a greater reduction in tinnitus distress after undertaking an ICBT intervention. The participants in this current trial represented those with higher levels of education as the majority had a university degree, college, or vocational training. When subgrouping those with tinnitus, Beukes et al. (56) suggested that the unique management pathways may be more suited for some tinnitus subgroups. Further work is required to identify which individuals with tinnitus are more suited for ICBT.

### Processes Involved in Intervention Delivery

All the participants who were assigned to the treatment were provided with access to the treatment program. However, several did not take the opportunity to engage with the material as 10 participants withdrew, and 30 participants never logged into the platform to access the intervention. Although attrition is similar to that of prior ICBT studies [mean of 14% (57)], engagement is lower than that previously reported in the trials in the United Kingdom [e.g., (16, 17)].

In addition, the initial modules were opened more than the final modules. The worksheet completion decreased during the later weeks of the intervention. Some participants indicated that they thought the intervention was too long which could be a contributing factor. Other participants found it helpful to have a comprehensive intervention. The intervention length and range of materials may, however, be a barrier for some. For those reading the modules, they rated the intervention highly in the terms of usefulness, applicability, and being able to understand the modules. The intervention dose was similar in the terms of guidance and delivery. The US intervention, however, had one additional module and more worksheets. Despite modifying it for ease of reading, the modules were opened by fewer participants compared with the participants of the United Kingdom who opened 74% of the recommended modules and 50% of the optional modules (34). When comparing these results with engagement by the population of the United Kingdom (16), stark differences are found. This earlier clinical trial indicated that the participants logged into the program on average 27 times compared with 8 times for the participants of the United States (23).

### Processes Involved in the Outcomes Obtained

Undertaking ICBT led to a significant reduction in tinnitus distress which was the primary aim of the intervention. The overall reduction with an effect size of *d* = 0.46 (*CI*: 0.14–0.77) was slightly lower than that compared with the pooled result of previous European ICBT trials of *d* = 0.50 (*CI*: 0.37–0.63) in the recent systematic review (57). These studies found a medium effect for ICBT reducing insomnia and a small effect for reducing anxiety and depression. The present study results varied as significant reductions being evident for the secondary outcomes for insomnia, tinnitus cognitions, and hearing disability but not for anxiety and depression. This may be related to those with significant levels of depression being excluded.

The compliance for completing the outcome measures was low, with 72% completion at the first time point, and dropping to 35% at the 1 year follow-up. This is lower than the previous ICBT in Europe, for example, the completing levels of 92 and 78% at post-intervention and 2 month follow-up for the participants from the United Kingdom (18). It may indicate that the population of the United States has other intervention needs or require additional motivation or incentives to complete the outcome measures. Satisfaction was lowest for motivation to complete, doing the worksheets, and how interesting the information was. Interestingly, the participants from the United Kingdom also rated these aspects the lowest (16, 34). The ways of increasing the motivation to do the intervention and worksheets are required. Overall satisfaction was lower than the ratings from the population of the United Kingdom where the majority of the scores were above 3/5 (16, 34). This may indicate the cultural differences or expectations from the interventions that may differ.

The facilitators identified were that the intervention was empowering, accessible, well-structured, and they were adequately supported while undertaking. Those thus undertaking the intervention found it very helpful and ways of getting more people to undertake the intervention are required.

### Processes Involved in the Trial Implementation

Adequate trial preparations were undertaken, such as assessing the intervention materials (20), the functionality of the platform (19), and doing a pilot study before commencing (21, 22). Although the implementation fidelity was high, the trial was run during the first wave of the COVID-19 pandemic. Due to the intervention being online the trial could, however, continue. It was apparent that some of the participants were unwell with COVID-19 and thus unable to engage as planned. Even after recovering, they found it difficult to do the program due to less energy. They were given more time to complete the program which impacted the intervention fidelity. Both the COVID-19 pandemic and virus have been shown to impact the tinnitus severity for some individuals (58). A subsection of participants (12%) in this study indicated that they had COVID-19 and 49% reported that the pandemic had negatively impacted their emotional state. It is likely that the pandemic and COVID-19 had a negative impact on the engagement in the intervention, but the extent of the impact is difficult to untangle. The participants in the control group had a weekly questionnaire to complete during the active intervention period without receiving the intervention. Some participants expressed a dislike of these questionnaires which may have impacted their subsequent engagement in the trial.

This process evaluation provides an opportunity for the participants to highlight the factors that made undertaking the intervention difficult. Personal parries, such as time barriers, the impact of the COVID-19 pandemic, lack of self-discipline, and other health problems were identified as barriers. The ways of increasing support to do the intervention should be sought. One idea may be involving significant others in the intervention process (59). This support may be motivational and help the intervention seem less burdensome. The intervention factors making the completion difficult included the length and number of tasks on the intervention, that tinnitus was heightened due to the focus on tinnitus during the intervention, and that some people sought a cure and not strategies to help them cope with the tinnitus. Such barriers can be reduced by ensuring the potential participants have a good understanding of exactly what the intervention entails. Modifying the intervention to ensure it is less time consuming but still comprehensive is required. The facilitators to the intervention's effectiveness were that it is empowering, accessible, and well-structured. The participants greatly valued the support they received from the guidance provided.

### Study Limitations and Future Directions

This evaluation was based on the barriers and facilitators identified by the participants completing the outcome measures. Although those not engaging were contacted by email, text, and phone, it was not always possible to reach them. This process evaluation would have benefited from including the views of those who did not engage or complete the outcome measures to truly reflect the barriers to participation. More effective ways of measuring engagement are required. Although it is possible to see if someone has opened a module, it is not possible to determine how much they have read, or how long they spent on the chapter. The outcomes measures used were all based on the clinical outcomes. For tinnitus, there may be more important or relevant outcomes not included that could have provided more insights. Future studies should investigate these, such as the intervention effectiveness on participation in the activities, impact on work, and relationships.

## Conclusions

This process evaluation has provided a broader understanding of the factors affecting recruitment and the research context. The impact of factors, such as social and family support should be considered (24–26). The aspects that contributed to the effectiveness of the intervention, such as the participants finding it empowering, accessible, and well-structured were identified. The barriers restricting engagement, such as the intervention length, time limitations, and low self-discipline levels need addressing. The results of this process evaluation should be implemented into further clinical trials to improve the reach, engagement, and outcomes obtained.

## Data Availability Statement

The datasets presented in this study can be found in online repositories. The names of the repository/repositories and accession number(s) can be found at: http://doi.org/10.6084/m9.figshare.13646012.

## Ethics Statement

Ethical approval was obtained from the Institutional Review Board at Lamar University, Beaumont, Texas, US (IRB-FY17-209). The patients/participants provided their written informed consent online to participate in this study.

## Author Contributions

The study was conceived by VM, GA, and EB. The study platform was provided by GA. The data collection and analysis were done by EB. EB drafted the manuscript. All the authors critically analyzed the full manuscript and approved the final version.

## Funding

This work was funded by the National Institute on Deafness and Communication Disorders (NIDCD) of the National Institute of Health (NIH) under the award number R21DC017214.

## Conflict of Interest

The authors declare that the research was conducted in the absence of any commercial or financial relationships that could be construed as a potential conflict of interest.

## Publisher's Note

All claims expressed in this article are solely those of the authors and do not necessarily represent those of their affiliated organizations, or those of the publisher, the editors and the reviewers. Any product that may be evaluated in this article, or claim that may be made by its manufacturer, is not guaranteed or endorsed by the publisher.
